# Aromatic L-amino Acid Decarboxylase (AADC) deficiency: results from an Italian modified Delphi consensus

**DOI:** 10.1186/s13052-021-00954-4

**Published:** 2021-01-21

**Authors:** Carlo Fusco, Vincenzo Leuzzi, Pasquale Striano, Roberta Battini, Alberto Burlina, the Delphi panel experts’ group, Vincenzo Belcastro, Donatella Capodiferro, Maria Alice Donati, Daniela Gueraldi, Francesca Manzoni, Alessandro Orsini, Pasquale Parisi, Francesco Pisani, Michele Sacchini, Alberto Spalice, Alberto Verrotti di Pianella, Maurizio Viri, Carlotta Spagnoli

**Affiliations:** 1 Child Neurology and Psychiatric Unit-Presidio Ospedaliero Santa Maria Nuova -AUSL-IRCCS di Reggio Emilia, Reggio Emilia, Italy; 2 Department of Paediatric Neuropsychiatry, Università La Sapienza, Rome, Italy; 3 Paediatric Neurology and Muscular Diseases Unit, IRCCS Istituto G. Gaslini, Genoa, Italy; 4 Department of Neurosciences, Rehabilitation, Ophthalmology, Genetics, Maternal and Child Health, University of Genova, Genoa, Italy; 5 Department of Developmental Neuroscience, Scientific Institute for Child and Adolescent Neuropsychiatry - IRCCS Stella Maris Foundation, Pisa, Italy; 6 Department of Experimental Medicine, University of Pisa, Pisa, Italy; 7 Division of Inborn Metabolic Disease, Department of Pediatrics, University Hospital Padua, Padova, Italy

**Keywords:** AADC deficiency, Aromatic L-amino acid decarboxylase deficiency, Metabolic disease, Neurometabolic disorder, Delphi consensus, Delphi method

## Abstract

**Background:**

Aromatic L-amino acid decarboxylase (AADC) deficiency is a rare and underdiagnosed neurometabolic disorder resulting in a complex neurological and non-neurological phenotype, posing diagnostic challenges resulting in diagnostic delay. Due to the low number of patients, gathering high-quality scientific evidence on diagnosis and treatment is difficult. Additionally, based on the estimated prevalence, the number of undiagnosed patients is likely to be high.

**Methods:**

Italian experts in AADC deficiency formed a steering committee to engage clinicians in a modified Delphi consensus to promote discussion, and support research, dissemination and awareness on this disorder. Five experts in the field elaborated six main topics, each subdivided into 4 statements and invited 13 clinicians to give their anonymous feedback.

**Results:**

100% of the statements were answered and a consensus was reached at the first round. This enabled the steering committee to acknowledge high rates of agreement between experts on clinical presentation, phenotypes, diagnostic work-up and treatment strategies. A research gap was identified in the lack of standardized cognitive and motor outcome data. The need for setting up an Italian working group and a patients’ association, together with the dissemination of knowledge inside and outside scientific societies in multiple medical disciplines were recognized as critical lines of intervention.

**Conclusions:**

The panel expressed consensus with high rates of agreement on a series of statements paving the way to disseminate clear messages concerning disease presentation, diagnosis and treatment and strategic interventions to disseminate knowledge at different levels. Future lines of research were also identified.

**Supplementary Information:**

The online version contains supplementary material available at 10.1186/s13052-021-00954-4.

## Background

Aromatic L-amino acid decarboxylase (AADC) deficiency is a rare autosomal recessive neurometabolic disorder characterized by a severe impairment of serotonin, dopamine, norepinephrine and epinephrine biosynthesis. Since its first description in 1990 [1], approximately 135 cases have been described worldwide [2]. Global prevalence is unknown, although estimates report predicted birth rates of 1:90,000 in the USA, 1:118,000 in Europe and 1:182,000 in Japan [3], while in an at-risk population with neurological deficits of unknown origin, an estimated prevalence of 1:9000 was documented [4].

The majority of patients are severely affected, with early-onset hypotonia (within the first year of life), severe/profound developmental delay (with no or limited motor attainments) and oculogyric crises. Additional common findings include dystonia, hypokinesia, and autonomic dysfunction (excessive sweating, nasal congestion, hypersalivation, temperature instability, ptosis, pupillary dysfunction, hypotension). Sleep disturbances, irritability, feeding and swallowing difficulties, vomiting can also represent prominent non-motor symptoms [2, 5, 6].

However, a milder phenotype, including spontaneous improvement during the second decade of life [7], with independent walking and feeding [8] and syndromic intellectual disability with autonomic dysfunction but no dystonia or oculogyric crises [9] has also been described.

Even though long-term outcome data are scanty, a significant childhood mortality risk has been suggested [2, 10], secondary to complications of pneumonia or acute events, at times in the context of oculogyric crises [2].

In the current lack of any approved therapy, clinicians’ priority is to identify the critical steps to take to improve the diagnostic rate and deliver the best care to AADC deficiency patients. To address potential lines of intervention enabling to achieve this goal, a Delphi consensus panel was organized by a group of Italian experts in AADC deficiency. The aims of this project were: a) to promote discussion between experts to improve knowledge on AADC deficiency; b) to give support to research, dissemination and actions to be undertaken locally to raise awareness on this rare disease; c) to reach a consensus between experts on the key interventions to set up to deliver better care and management to patients with AADC deficiency.

## Methods

### Modified Delphi method

The Delphi method is an iterative investigation method aiming to reach the best estimate of consensus and to define standards, therapeutic or management procedures, and to elaborate guidelines or provide a recommendation on controversial topics. Each expert freely, independently and anonymously gives his/her opinion through one or more rounds of discussion. After each round, a summary of the experts’ answers and their rationale is provided. The process ends when an agreement has been reached on the discussed topics. For this study, we used the modified Delphi technique, involving a set of carefully selected items drawn from synthesized reviews of the literature rather than open-ended questions, providing a highly structured and transparent process to obtain feedback [11–14].

### Steering committee

The steering committee included 5 medical doctors (one neurologist, three child neurologists and one paediatrician) with special expertise in treating patients with AADC.

### Delphi participants

The voting panel was composed of 13 medical doctors, with clinical experience in managing patients with AADC, divided as follows: one neurologist, five paediatricians, 5 child neurologists and 2 specialized in both neurology and paediatrics (the full list of participants is reported in the Additional file 1).

### Selection of Delphi questionnaire statements

Based on a careful review of the literature performed before the first meeting (October 2019), the steering committee selected 6 controversial topics: clinical manifestations, clinical phenotypes, diagnostic work-up, treatments, patients’ associations and follow-up. For each of them, one of the authors declined four items on which the participating experts expressed their level of agreement.

### Delphi rounds

After validation of the statements by external reviewers, an invitation letter by the steering committee was sent to the 13 participants outlining the study aims and procedure. Panel members were asked to fill in an online questionnaire through a secure web platform, available at the following link www.consensusdelphi-aadcd.it between 30th March and 10th April 2020. To reduce the risk of bias or influence by other specialists’ opinions, the answers were collected anonymously.

Clinicians expressed their level of agreement or disagreement on each statement by using a 5-point-Likert-type scale (1 = strongly disagree, 2 = disagree, 3 = somewhat agree, 4 = agree, and 5 = strongly agree). The absolute number and percentage of participants who scored each item as 1 or 2 (disagreement) or as 3, 4 or 5 (agreement) were calculated. The consensus was considered to be reached when the sum for disagreement or agreement was ≥66%.

Based on a summary of the scores received, all items were ranked by the steering committee in a single Delphi round. Responses from the experts were summarized descriptively (numbers, percentages) and graphically to identify outliers. Finally, results were discussed by the steering committee and a series of consensus-based recommendations were finalized. No second-round vote was performed since there was no statement with controversial answers in the first round.

**Fig. 1:**
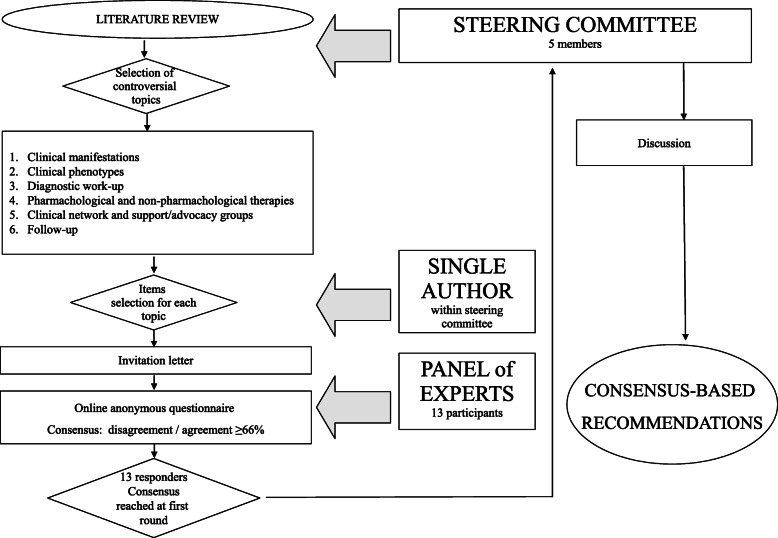
Fow-chart representing the process leading to finalization of recommendations

The whole process leading to the finalization of recommendations has been represented graphically in Fig. 1.

## Results

Six main areas of discussion were selected: AADC clinical manifestations and phenotypes, diagnostic work-up, therapy, clinical network and advocacy groups, and follow-up.

As the first round of the survey, 13 participants completed the questionnaire for all 6 statements. The consensus was achieved for all statements at first round (view the Additional documentation 1 for Delphi Consensus Results and Statements).

### Statement 1: clinical manifestations

The consensus was complete on the high phenotypic variability (in part related to age at onset) and the predominating presentation with autonomic dysfunction associated with movement disorders such as hypotonia/hypokinesia (very early presentations) and/or dyskinesias and developmental delay (100% agreement). An agreement was almost complete on the presence of significant differences in clinical characteristics between early-onset and adult-onset cases (85% consensus, two experts disagreed) and on the onset of symptoms within the first year of life (although possibly mild), regardless of clinical severity or phenotypic variability, in all cases (92% consensus, one expert disagreed).

### Statement 2: clinical phenotypes

The experts expressed complete consensus (100%) on all of the statements regarding clinical phenotypes: the need to raise awareness on underestimated symptoms (i.e. oculogyric crises, dystonia or dyskinesia); non-diabetic hypoglycemic crises not always being recognized as a sign of metabolic dysfunction in AADC deficiency; the usefulness of recognizing common AADC symptoms in association with hypoglycemic crises and non-neurological symptoms (diarrhea, gastro-oesophagal reflux, feeding difficulties, nasal congestion) for early diagnosis; and on epileptic encephalopathy being a rare and atypical presentation.

### Statement 3: diagnostic work-up

There was full agreement on the usefulness of considering whole-exome sequencing in the diagnostic work-up of patients with adult-onset forms with mild phenotype and on the need to consider dosing plasma enzymatic AADC activity in cases with doubtful diagnosis if CSF neurotransmitters show non-significant abnormalities (100% consensus). There was nearly complete consensus on the need to analyze the CSF neurotransmitters profile as the first diagnostic step in suspected cases (92%, one expert disagreed). The consensus was also reached on the usefulness of dosing venous 3-O-methyl-dopa (3-OMD) levels as a valid, cheap and readily available screening tool to decide whether to begin the diagnostic work-up (85% consensus, one expert disagreed and one strongly disagreed). 3-OMD concentration is increased in dried blood spots of AADC deficient patients and hence is a candidate biomarker for the pre-symptomatic diagnosis if implemented in newborn screening programs.

### Statement 4: pharmacological and non-pharmacological therapies

The consensus was complete (100%) for all statements. Specifically, the experts agreed on considering dopamine agonists, MAO inhibitors, and vitamin B6 as the current first therapeutic choice in patients with AADC deficiency, on the common use of anticholinergic drugs for symptomatic treatment of the movement disorder and of benzodiazepines in specific cases (i.e. dystonic status or oculogyric crises). Moreover, all the experts agreed on the need for a multidisciplinary approach in AADC deficiency patients’ follow-up. Finally, early diagnosis is considered necessary by all experts to expect the best improvements from AAV vector-based gene therapy even if it is not required to be eligible for AAV vector-based gene therapy (currently seeking approval by regulatory agencies).

### Statement 5: clinical network and support/advocacy groups

All the experts agreed on the need to raise awareness on AADC deficiency, by involving the scientific societies operating in the pediatric, child neurology and neurometabolic fields, by involving working groups dedicated to rare diseases throughout Italy and by creating a dedicated working group for AADC deficiency (100% consensus). There was almost complete agreement on the need to consider creating an association of patients through a clinical network (92% consensus, one expert disagreed).

### Statement 6: follow-up

There was complete consensus on the need to evaluate cognitive and neuropsychological functions with standardized, age-appropriate scales, to correctly characterize the movement disorder phenotype by using age-appropriate, standardized scales and to constantly apply these scales in patients’ follow-up to evaluate response to treatment and cognitive and motor outcomes. Finally, there was also full agreement on the need to evaluate cognitive and motor improvement associated with development and improvements in movement disorder severity following drug or gene therapy (100% consensus).

## Discussion

By using a modified Delphi method, a panel of experts reached consensus on clinical, diagnostic and therapeutic cornerstones of AADC deficiency management, and clinically-relevant research gaps. Key lines of intervention to sensitize referring clinicians and the general audience at a national and local level were also identified. This initiative of national experts has identified a series of clinical features possibly promoting AADC diagnosis even among non-specialist physicians and provided relevant expert insights into the main barriers to early diagnosis.

Full agreement was reached on the high phenotypic variability and on the typical association of neurological and extra-neurological symptoms, which can make diagnosis challenging. The panel agreed that despite the presence of significant differences in clinical characteristics between early-onset and adult-onset cases, true clinical onset always occurs in infancy, regardless of severity. However, some of the symptoms and signs might be overlooked or not attributed to AADC deficiency, resulting in significant diagnostic delay [15]. Especially in early-onset forms, unspecific neurological symptoms and signs such as developmental delay and hypotonia/hypokinesia can be either underestimated or interpreted as of neuromuscular origin (with a misdiagnosis of myasthenia [2, 16]).

The panel acknowledged an urgent need to raise awareness on underestimated neurological symptoms such as oculogyric crises, dystonia or dyskinesia, and on non-neurological symptoms, such as non-diabetic hypoglycemic crises, often not correctly attributed to metabolic dysfunction in AADC deficiency [6, 17], or vegetative symptoms (diarrhea, gastroesophageal reflux, feeding difficulties, nasal congestion). These latter are often poorly described in the literature, even though they can become prominent and disabling since childhood [18], likely resulting in referral to specialists unfamiliar with inherited neurotransmitters disorders, and – consequently— in diagnostic delay.

Regarding diagnostic tests, while the consensus was unanimous in considering whole-exome sequencing in the diagnostic work-up of patients with mild, adult-onset (less specific) phenotypes, and on the need to consider dosing plasma enzymatic AADC activity if CSF neurotransmitters show non-significant abnormalities (100% consensus), the agreement was still high, although not complete, on the need to analyze CSF neurotransmitters as the first diagnostic step in suspected cases (one expert disagreed) and on dosing blood 3-OMD as a valid screening tool at the beginning of the diagnostic work-up (one expert disagreed and one strongly disagreed). Based on the literature review and the recent consensus guidelines [17], lumbar puncture (showing a typical profile with low levels of 5-HIAA, HVA and MHPG, increased levels of 3-OMD, L-dopa and 5-HTP, and normal pterins), molecular diagnosis and AADC activity in plasma is strongly recommended, and two out of three criteria are required to confirm the diagnosis [17]. Additionally, recent studies applying dried blood spot 3-OMD dosing have shown a positive predictive value of 100% in newborns [19] and a > 15-fold increase in neonates and children with AADC deficiency compared to controls [20], thus making this test a suitable and readily available screening tool to start the diagnostic workup [4].

Although the quality of the available evidence is poor and no specific therapy has yet been approved, there was full agreement on therapeutic aspects, as the clinical management of AADC deficiency is based on symptomatic treatment for which recommendations have been provided in 2017 [17]. However, it must be emphasized that the efficacy of symptomatic therapy is disappointing in the majority of cases [15], although some researches have suggested clinical improvement in milder forms [6, 7]. Finally, early diagnosis is considered necessary by all experts to expect the best improvements from AAV vector-based gene therapy even if it is not required to be eligible for AAV vector-based gene therapy (currently seeking approval by regulatory agencies).

Identified cardinal interventions to raise awareness on this disorder in Italy include involvement of multidisciplinary scientific societies; involvement of working groups focusing on rare diseases; creation of an AADC working group; creation of a patients’ association through a clinical network. Although government-based policies on rare diseases have been promoted worldwide, and especially in Europe [21], actively involving patients’ communities and clinicians at a local level would be a primary target to increase knowledge on presentation and correct diagnostic and therapeutic management. Comparing the estimated prevalence of AADC deficiency with the number of reported patients makes evident that the majority of affected individuals are still undiagnosed [3]. Recent research focusing on the information needs of physicians in Belgium documented perceived lack of academic education on rare diseases [22]. Lack of awareness among physicians working in the community services might result in the clinical suspicion of a rare disorder never being raised, or in the family being referred to a high number of different specialists before the correct diagnosis is formulated.

The creation of an AADC deficiency patients association has been identified as a strategy to promote awareness on this disorder, based on a body of evidence documenting a positive role of advocacy groups in addressing difficulties in accessing timely diagnosis and appropriate treatment in rare diseases [21]. Patient advocacy groups can also facilitate research, helping with patient recruitment, research funding, patient assistance programs, and facilitation of communication [23].

Finally, the experts identified an important knowledge gap in the lack of follow-up data on cognition and motor function examined with standardized scales [17]. These are necessary to identify specific profiles (if present), to gain deeper insight into the natural history of the disorder, to assess the effectiveness of therapeutic interventions and to increase the reproducibility of results in clinical research.

In this study, we utilized a modified Delphi technique to establish consensus from an expert panel. The use of expert opinion was necessary since data from randomized clinical trials are lacking due to the small number of patients. Research into rare diseases faces unique challenges, including difficulties in establishing diagnoses, in recruiting subjects into research studies, the paucity of expert centres, and lack of awareness among treating physicians [24].

## Conclusions

We showed high rates of agreement on a series of statements paving the way to the dissemination of clear clinical messages concerning disease presentation, diagnosis and treatment and strategic interventions to disseminate knowledge at different levels. Potential future lines of research were also identified. This research aimed to set out critical lines of intervention to achieve increased scientific knowledge and empowered diagnostic suspicion, intending to deliver better care to Italian patients with AADC deficiency.

## Supplementary Information

**Additional file 1.**

## Abbreviations

AADC: Aromatic L-amino acid decarboxylase
CSF: Cerebrospinal fluid
3-OMD: 3-O-methyl-dopa
MAO: Monoamine oxidase
AAV: Adeno-Associated Virus
HIAA: 5-Hydroxyindoleacetic Acid
HVA: homovanillic acid
MHPG: 3-Methoxy-4-hydroxyphenylglycol
5-HTP: 5-hydroxytryptophan
