# Supplementary material for: Aromatic L-amino Acid Decarboxylase (AADC) deficiency: results from an Italian modified Delphi consensus
Source: Ital J Pediatr. 2021 Jan 21;47:13. doi: 10.1186/s13052-021-00954-4 (PMC7819234; doi:10.1186/s13052-021-00954-4)
Supplement: Supplementary file 1 — Additional file 1. [file 13052_2021_954_MOESM1_ESM.pdf]

Consensus Delphi
"AADCd Aromatic L-amino acid decarboxylase deficiency”

Belcastro Vincenzo	Nuovo Ospedale S. Anna di Como
Capodiferro Donatella	Ospedale SS. Annunziata di Taranto
Donati Maria Alice	AOU Meyer di Firenze
Gueraldi Daniela	AOU di Padova
Manzoni Francesca	AOU di Padova
Orsini Alessandro	AOU di Pisa
Parisi Pasquale	Ospedale Sant’Andrea di Roma
Pisani Francesco	Università degli Studi di Parma
Sacchini Michele	AOU Meyer di Firenze
Spagnoli Carlotta	ASMN di Reggio Emilia
Spalice Alberto	Università La Sapienza di Roma
Verrotti di Pianella Alberto	Università degli Studi dell’Aquila
Viri Maurizio	Ospedale Maggiore di Novara

Statement 1. AADC clinical manifestations:

	1	2	3	4	5	TOT
1.1 There is high phenotypic variability, partly related to age at onset.	0	0	7	6	0	13
	0%		100%			100%
1.2 Predominating clinical manifestations at presentation in early-onset forms are autonomic, associated with hypotonia/movement disorder, and developmental delay.	0	0	1	6	6	13
	0%		100%			100%
1.3 There are significant differences in clinical characteristics between early-onset and adult-onset cases.		2	3	5	3	13
	15%		85%			100%
1.4 Regardless of clinical severity and phenotypic variability, onset symptoms are always present since the first year of age, although they can be mild.		1	5	5	2	13
	8%		92%			100%

Statement 2. Clinical phenotypes:

	1	2	3	4	5	TOT
2.1 It is necessary to raise awareness on underestimated symptoms, such as oculogyric crises, dystonia or dyskinesia.			2	4	7	13
	0%		100%			100%
2.2 The occurrence of hypoglycemic crises (non-diabetes-related) is not always recognized as a sign of metabolic dysfunction in AADC deficiency.			4	4	5	13
	0%		100%			100%
2.3 The identification of common AADC symptoms associated with recognition of hypoglycemic crises and additional non-neurological symptoms (dyarrhea, gastro-oesophageal reflux, feeding difficulties, nasal congestion) enables early diagnosis.			2	7	4	13
	0%		100%			100%
2.4 In early-onset forms, presentation with epileptic encephalopathy is rare and does not represent a typical clinical feature.			1	8	4	13
	0%		100%			100%

Statement 3. Diagnostic work-up:

	1	2	3	4	5	TOT
3.1 1 The analysis of CSF neurotransmitters profile should represent the first diagnostic step.		1	1	6	5	13
	8%		92%			100%
3.2 In adult-onset forms with mild phenotype (for which diagnosis is even harder) whole exome sequencing should be considered in the diagnostic work-up.			4	5	4	13
	0%		100%			100%
3.3 Based on the available evidence, venous 3-O-methyl-dopa (3OMD) levels represent a cheap, valid and readily available biomarker on which to base decision to begin diagnostic work-up for AADCd	1	1	3	5	3	13
	15%		85%			100%
3.4 Dosing plasma enzymatic AADC activity should be considered in cases with doubtful diagnosis if CSF neurotransmitters show non-significant abnormalities.			5	3	5	13
	0%		100%			100%

Statement 4. Regarding pharmacological and non-pharmacological therapies:

	1	2	3	4	5	TOT
4.1 Dopamine agonists, MAO inhibitors, and vitamin B6 currently represent the first therapeutic choice in AADC patients.			1	4	8	13
	0%		100%			100%
4.2 Anticholinergic drugs are commonly used for the symptomatic treatment of movement disorder. In some cases, for example status dystonicus or oculogyric crises, administration of benzodiazepines can become necessary.			3	6	4	13
	0%		100%			100%
4.3 The clinical management should be offered by a multi-disciplinary team (physical and occupational therapist, gastroenterologist, speech and language therapist, psychologist, clinical geneticist).			2	3	8	13
	0%		100%			100%
4.4 Early diagnosis is necessary in order to access gene therapy based on AAV vector, which is currently seeking approval by regulatory agencies.			2	4	7	13
	0%		100%			100%

Statement 5. Clinical network and support/advocacy groups:

	1	2	3	4	5	TOT
5.1 Awareness on AADC deficiency should be spread by involving scientific societies operating in pediatrics, child neurology and neurometabolics.			2	3	8	13
	0%		100%			100%
5.2 It is useful to increase awareness even outside clinical-scientific contexts by involving working groups dedicated to rare diseases in various Italian regions.			3	2	8	13
	0%		100%			100%
5.3 Based on the low number of patients in Italy, it is necessary to develop a dedicated working group for AADCd.			3	2	8	13
	0%		100%			100%
5.4 It is necessary to consider the opportunity to create a patients' association by means of a clinical network.		1	3	2	7	13
	8%		92%			100%

Statement 6. Follow-up:

	1	2	3	4	5	TOT
6.1 Cognitive and neuropsychological evaluation (appropriate for patient's age) should be performed by using standardized scales.			2	4	7	13
	0%		100%			100%
6.2 It is necessary to correctly characterize the associated movement disorder by means of standardized scales, according to patient's age (MD CRS 0-3; MD CRS 4-18R).			2	4	7	13
	0%		100%			100%
6.3 Standardized testing should be constantly applied during follow-up in order to evaluate treatment response, and cognitive and motor outcomes by using appropriate scales, as these data are lacking from the literature.			2	4	7	13
	0%		100%			100%
6.4 Improvements associated with cognitive and motor development and movement disorder severity after drug or gene therapy should be monitored by administering standardized scales.			2	3	8	13
	0%		100%			100%
